# Bipolar Disorder and Alcoholism

**Published:** 2002

**Authors:** Susan C. Sonne, Kathleen T. Brady

**Affiliations:** Susan C. Sonne, PharmD, is a research assistant professor of psychiatry and behavioral sciences and clinical assistant professor of pharmacy practice, and Kathleen T. Brady, M.D., Ph.D., is a professor of psychiatry and behavioral sciences, both at the Medical University of South Carolina, Center for Drug and Alcohol Programs, Charleston, South Carolina

**Keywords:** comorbidity, manic-depressive psychosis, AODD (alcohol and other drug dependence), alcoholic beverage, prevalence, genetic linkage, disease onset, disease course, diagnosis, drug therapy, lithium, valproate, naltrexone, patient compliance, psychosocial treatment method, literature review

## Abstract

Bipolar disorder and alcoholism commonly co-occur. Multiple explanations for the relationship between these conditions have been proposed, but this relationship remains poorly understood. Some evidence suggests a genetic link. This comorbidity also has implications for diagnosis and treatment. Alcohol use may worsen the clinical course of bipolar disorder, making it harder to treat. There has been little research on the appropriate treatment for comorbid patients. Some studies have evaluated the effects of valproate, lithium, and naltrexone, as well as psychosocial interventions, in treating alcoholic bipolar patients, but further research is needed.

Bipolar disorder and alcoholism co-occur at higher than expected rates. That is, they co-occur more often than would be expected by chance and they co-occur more often than do alcoholism and unipolar depression. This article will explore the relationship between these disorders, focusing on the prevalence of this comorbidity, potential theoretical explanations for the high rates of comorbidity, effects of comorbid alcoholism on the course and features of bipolar disorder, diagnostic issues, and treatment of comorbid patients.

Bipolar disorder, often called manic depression, is a mood disorder that is characterized by extreme fluctuations in mood from euphoria to severe depression, interspersed with periods of normal mood (i.e., euthymia). Bipolar disorder represents a significant public health problem, which often goes undiagnosed and untreated for lengthy periods. In a survey of 500 bipolar patients, 48 percent consulted 5 or more health care professionals before finally receiving a diagnosis of bipolar disorder, and 35 percent spent an average of 10 years between the onset of illness and diagnosis and treatment (Lish et al. 1994). Bipolar disorder affects approximately 1 to 2 percent of the population and often starts in early adulthood.

There are a number of disorders in the bipolar spectrum, including bipolar I disorder, bipolar II disorder, and cyclothymia. Bipolar I disorder is the most severe; it is characterized by manic episodes that last for at least a week and depressive episodes that last for at least 2 weeks. Patients who are fully manic often require hospitalization to decrease the risk of harming themselves or others. People can also have symptoms of both depression and mania at the same time. This mixed mania, as it is called, appears to be accompanied by a greater risk of suicide and is more difficult to treat. Patients with 4 or more mood episodes within the same 12 months are considered to have rapid cycling bipolar disorder, which is a predictor of poor response to some medications. Bipolar II disorder is characterized by episodes of hypomania, a less severe form of mania, which lasts for at least 4 days in a row and is not severe enough to require hospitalization. Hypomania is interspersed with depressive episodes that last at least 14 days. People with bipolar II disorder often enjoy being hypomanic (due to elevated mood and inflated self-esteem) and are more likely to seek treatment during a depressive episode than a manic episode. Cyclothymia is a disorder in the bipolar spectrum that is characterized by frequent low-level mood fluctuations that range from hypomania to low-level depression, with symptoms existing for at least 2 years (American Psychiatric Association [APA] 1994).

Alcohol dependence, also known as alcoholism, is characterized by a craving for alcohol, possible physical dependence on alcohol, an inability to control one’s drinking on any given occasion, and an increasing tolerance to alcohol’s effects (APA 1994). Approximately 14 percent of people experience alcohol dependence at some time during their lives (Kessler et al. 1997). It often starts in early adulthood. Criteria for a diagnosis of alcohol abuse, on the other hand, do not include the craving and lack of control over drinking that are characteristic of alcoholism. Rather, alcohol abuse is defined as a pattern of drinking that results in the failure to fulfill responsibilities at work, school, or home; drinking in dangerous situations; and having recurring alcohol-related legal problems and relationship problems that are caused or worsened by drinking (APA 1994). The lifetime prevalence of alcohol abuse is approximately 10 percent (Kessler et al. 1997). Alcohol abuse often occurs in early adulthood and is usually a precursor to alcohol dependence (APA 1994).

## Prevalence of Comorbidity

Several studies have reported an association between alcoholism and mood disorders. To date, there have been two large epidemiological studies of psychiatric disorders: the National Institute of Mental Health’s Epidemiologic Catchment Area (ECA) study (Regier et al. 1990) and the National Comorbidity Survey (NCS) (Kessler et al. 1996). The ECA study (Regier et al. 1990) revealed that 60.7 percent of people with bipolar I disorder had a lifetime diagnosis of a substance use disorder (i.e., an alcohol or other drug use disorder); 46.2 percent of those with bipolar I disorder had an alcohol use disorder; and 40.7 percent had a drug abuse or dependence diagnosis (the percentages of people with alcohol use disorders and drug abuse disorders do not add to 100 due to overlap). Forty-eight percent of people with bipolar II disorder had a substance use disorder, 39.2 percent had an alcohol use disorder, and 21 percent had a drug abuse or dependence diagnosis (these figures reflect overlap, as above.) As shown in the table, alcohol dependence was twice as likely to co-occur in people with bipolar spectrum disorders than in those with unipolar depression (i.e., depression without mania). It is also noteworthy that bipolar disorder was more likely to occur with alcohol dependence than with alcohol abuse (see table). As part of the ECA study, Helzer and Przybeck (1988) found that mania (i.e., bipolar I disorder) and alcohol use disorders are far more likely to occur together (i.e., 6.2 times more likely) than would be expected by chance. Of all other psychiatric diagnoses investigated in this study, only antisocial personality disorder was more likely to be related to alcoholism than mania. The findings of the NCS with regard to the comorbidity of mood disorders and alcoholism were very similar.

**Table:** Comorbid Mood Disorders* and Substance Abuse

	Any substance abuse or dependence (%)	Alcohol dependence (%)	Alcohol abuse (%)
Any Mood Disorder	32.0	4.9	6.9
Any Bipolar Disorder	56.1	27.6	16.1
Bipolar I	60.7	31.5	14.7
Bipolar II	48.1	20.8	18.4
Unipolar Depression	27.2	11.6	5.0

NOTES:

* Mood disorders include depression and bipolar disorder.

Bipolar disorder, or manic depression, is characterized by extreme mood swings.

Bipolar I disorder is the most severe bipolar disorder.

Bipolar II disorder is less severe.

Unipolar depression is depression without manic episodes.

SOURCE: Data reported in the table are based on findings of the Epidemiologic Catchment Area study (Regier et al. 1990).

## Possible Explanations for Comorbidity

Although researchers have proposed explanations for the strong association between alcoholism and bipolar disorder, the exact relationship between these disorders is not well understood. One proposed explanation is that certain psychiatric disorders (such as bipolar disorder) may be risk factors for substance use. Alternatively, symptoms of bipolar disorder may emerge during the course of chronic alcohol intoxication or withdrawal. For example, alcohol withdrawal may trigger bipolar symptoms. Still other studies have suggested that people with bipolar disorder may use alcohol during manic episodes in an attempt at self-medication, either to prolong their pleasurable state or to sedate the agitation of mania. Finally, other researchers have suggested that alcohol use and withdrawal may affect the same brain chemicals (i.e., neurotransmitters) involved in bipolar illness, thereby allowing one disorder to change the clinical course of the other. In other words, alcohol use or withdrawal may “prompt” bipolar disorder symptoms (Tohen et al. 1998). It remains unclear which if any of these potential mechanisms is responsible for the strong association between alcoholism and bipolar disorder. It is very likely that this relationship is not simply a reflection of cause and effect but rather that it is complex and bidirectional. Genetic factors may also play a role, as described below.

### Familial Risk of Bipolar Disorder and Alcoholism

The role of genetic factors in psychiatric disorders has received much attention recently. Some evidence is available to support the possibility of familial transmission of both bipolar disorder and alcoholism (Merikangas and Gelernter 1990; Berrettini et al. 1997). Common genetic factors may play a role in the development of this comorbidity, but this relationship is complex (Tohen et al. 1998). Preisig and colleagues (2001) conducted a family study of mood disorders and alcoholism by evaluating 226 people with alcoholism with and without a mood disorder as well as family members of those people. The researchers found that there was a greater familial association between alcoholism and bipolar disorder (odds ratio of 14.5) than between alcoholism and unipolar depression (odds ratio of 1.7). These findings have implications for prevention and treatment. A positive family history of bipolar disorder or alcoholism is an important risk factor for offspring.

## Issues Surrounding the Treatment of Comorbid Bipolar Disorder and Alcoholism

This section examines some of the issues to consider in treating comorbid patients, and a subsequent section reviews pharmacologic and psychotherapeutic treatment approaches.

### Alcoholism’s Effect on Comorbid Bipolar Disorder

A growing number of studies have shown that substance abuse, including alcoholism, may worsen the clinical course of bipolar disorder. Sonne and colleagues (1994) evaluated the course and features of bipolar disorder in patients with and without a lifetime substance use disorder. They found that compared to non-substance abusers, substance-abusing bipolar patients were more likely to have frequent hospitalizations for affective symptoms, earlier onset of bipolar disorder, more rapid cycling, and more mixed mania (the latter two considered to be the most severe, treatment-resistant forms of bipolar disorder). Keller and colleagues (1986) compared patients who had pure depression or pure mania with patients who had mixed or rapid cycling bipolar disorder and found that a higher percentage of patients with mixed or rapid cycling bipolar disorder had concurrent alcoholism (13 percent) and that these patients had a slower recovery from the bipolar disorder. Although this association does not necessarily indicate that alcoholism worsens bipolar symptoms, it does point out the relationship between them. A comparison of patients with bipolar disorder and a coexisting substance use disorder with others who had bipolar disorder alone found that those with comorbid substance use disorders had an earlier age of onset for their mood disorder, were more likely to be male, had more comorbid psychiatric disorders in addition to bipolar disorder, and were significantly more likely to have mixed mania at the time of interview (Sonne and Brady 1999*b*).

Although research suggests that alcohol and other drug abuse may worsen the course of bipolar disorder, some data indicate that patients with bipolar disorder and alcoholism do better in substance abuse treatment than alcoholic patients with other mood disorders. O’Sullivan and colleagues (1988) found that alcoholics with bipolar disorder functioned better during a 2-year followup period than did primary alcoholics (i.e., those without comorbid mood disorders) or alcoholics with unipolar depression. This suggests that bipolar patients may use alcohol primarily as a means to medicate their affective symptoms, and if their bipolar symptoms are adequately treated, they are able to stop abusing alcohol. Hasin and colleagues (1989) found that patients with bipolar II disorder were likely to have an earlier remission from alcoholism compared with patients with schizoaffective disorder or bipolar I disorder. Researchers have also proposed that the presence of mania may precipitate or exacerbate alcoholism (Hasin et al. 1985).

In conclusion, it appears that alcoholism may adversely affect the course and prognosis of bipolar disorder, leading to more frequent hospitalizations. In addition, patients with more treatment-resistant symptoms (i.e., rapid cycling, mixed mania) are more likely to have comorbid alcoholism than patients with less severe bipolar symptoms. If left untreated, alcohol dependence and withdrawal are likely to worsen mood symptoms, thereby forming a vicious cycle of alcohol use and mood instability. However, some data indicate that with effective treatment of mood symptoms, patients with bipolar disorder can have remission of their alcoholism.

### Order of Onset

An important factor in studying the influence of one comorbid disorder on another is the order of onset of the two disorders. A mood disorder that occurs prior to the onset of another psychiatric disorder is called a primary affective disorder. Secondary affective disorders occur after the onset of other psychiatric disorders. Feinman and Dunner (1996) conducted a retrospective chart review of three groups of patients:

1. Those with primary bipolar disorder with no history of substance abuse (primary group), with 103 patients
2. Those with primary bipolar disorder complicated by substance abuse, which began after the onset of bipolar disorder (complicated group), with 35 patients
3. Those with bipolar disorder that came after the onset of substance abuse (secondary group), with 50 patients.

The researchers found that patients in the complicated group had a significantly earlier age of onset of bipolar disorder than the other groups. They also found that the complicated and secondary groups had higher rates of suicide attempts than did the primary group. Preisig and colleagues (2001) also reported that the onset of bipolar disorder tended to precede that of alcoholism. They concluded that this finding is in accordance with results of clinical studies that suggest alcoholism is often a complication of bipolar disorder rather than a risk factor for it.

In a 5-year followup study, Winokur and colleagues (1995) evaluated a group of bipolar patients with and without alcoholism. In the alcoholic patients, bipolar illness and alcoholism were categorized as being either primary or secondary. The patients with primary alcoholism had significantly fewer episodes of mood disorder at followup, which may suggest that these patients had a less severe form of bipolar illness.

Thus, there is growing evidence that the presence of a concomitant alcohol use disorder may adversely affect the course of bipolar disorder, and the order of onset of the two disorders has prognostic implications. Specifically, bipolar patients with secondary alcoholism may be better able to stop drinking if their bipolar illness is adequately treated; and, conversely, bipolar patients with primary alcoholism (alcoholism occurs first) may be better able to control their mood symptoms if they are able to stop drinking.

### Comorbidity and Diagnostic Issues

Almost every alcoholic will report having mood swings. It is very important to distinguish these alcohol-induced symptoms from actual bipolar disorder. However, diagnosing bipolar disorder in the face of alcohol abuse can be difficult because alcohol use and withdrawal, particularly with chronic use, can mimic nearly any psychiatric disorder. Alcohol intoxication can produce a syndrome indistinguishable from mania or hypomania, characterized by euphoria, increased energy, decreased appetite, grandiosity, and sometimes paranoia. However, these alcohol-induced manic symptoms generally occur only during active alcohol intoxication, which makes them fairly easy to differentiate from mania associated with bipolar I disorder.

Still, alcoholic patients going through alcohol withdrawal may appear to have depression. Depression is a key symptom of withdrawal from several substances of abuse, and studies have demonstrated that symptoms of withdrawal-related depression may persist for 2 to 4 weeks (Brown and Schuckit 1988). Because of this phenomenon, it is likely that observation during lengthier periods of abstinence (i.e., continued observation following the withdrawal stage) is important for the diagnosis of depression as compared with mania.

Bipolar II disorder and cyclothymia are even more difficult to reliably diagnose because of the more subtle nature of the psychiatric symptoms. Because of the diagnostic difficulties, it may be that this diagnostic group is often overlooked. Although these less severe forms of bipolar disorder may not be as disruptive as bipolar I disorder, it is still important to recognize and treat them in order to break the potential cycle of mood problems leading to substance use, which leads to a worsening of mood symptoms, which in turn may worsen the substance abuse, leading to even worse mood symptoms.

As a general rule, it seems appropriate to diagnose bipolar disorder if the symptoms clearly occur before the onset of the alcoholism or if they persist during periods of sustained abstinence. The adequate amount of abstinence for diagnostic purposes has not been clearly defined. Family history and severity of symptoms should also factor into diagnostic considerations. Given that bipolar disorder and substance abuse co-occur so frequently, it also makes sense to screen for substance abuse in people seeking treatment for bipolar disorder.

## Treatment of Comorbid Bipolar Disorder and Alcoholism

In spite of the significant prevalence of comorbid alcoholism and bipolar disorder, there is little published data on specific pharmacologic and psychotherapeutic treatments for bipolar disorder in the presence of alcoholism. The medications most frequently used for treating bipolar disorder are the mood stabilizers lithium and valproate. As stated previously, preliminary evidence suggests that alcoholic bipolar patients may have more rapid cycling and more mixed mania than other bipolar patients. There is also evidence to suggest that these subtypes of bipolar disorder have different responses to medications (Prien et al. 1988), which would help provide a rationale for the choice of agents in the alcoholic bipolar patient. Available research on the use of lithium, valproate, and naltrexone for comorbid patients is reviewed below.

### Lithium

Lithium has been the standard treatment for bipolar disorder for several decades. Unfortunately, several studies have reported that substance abuse is a predictor of poor response of bipolar disorder to lithium. More specifically, as stated previously, compared to non-substance abusers, alcoholics appear to be at greater risk for developing mixed mania and rapid cycling. Researchers have found that patients with mixed mania respond less well to lithium than patients with the nonmixed form of the disorder (Prien et al. 1988). This suggests that lithium may not be the best choice for a substance-abusing bipolar patient. However, in a 6-week trial of lithium versus placebo in 25 adolescents with bipolar disorder and secondary substance dependence, Geller and colleagues (1998) found a significant reduction in positive urine tests for substances of abuse and significant improvement in psychiatric symptoms. This suggests that lithium may be a good choice for adolescent substance abusers. The presence of bipolar subtypes was not addressed in this study, so it is not clear if these adolescents had the subtypes of bipolar illness that are more difficult to treat.

### Valproate

In 1998, the anticonvulsant Depakote^®^ (also called divalproex sodium, or valproate) was approved by the Food and Drug Administration (FDA) for the initial treatment of manic episodes associated with bipolar disorder. Numerous studies have concluded that patients with mixed or rapid cycling bipolar disorder are more likely to respond to anticonvulsant medications than to lithium (Bowden 1995). Because, as stated previously, bipolar patients with concomitant alcoholism appear to have more mixed or rapid cycling bipolar disorder than do bipolar patients who are not alcoholic, alcoholic bipolar patients may also respond better to anticonvulsant medications (e.g., valproate) than to lithium therapy. In fact, in an open-label study (i.e., a study in which all participants receive the experimental treatment), Brady and colleagues (1995) found valproate to be safe and effective in nine mixed-manic bipolar patients with concurrent substance dependence (primarily alcohol dependence) who previously had either not tolerated lithium or not responded to it. Similarly, Albanese and coworkers (2000) reported on 20 patients treated with divalproex sodium and found that even at fairly low doses divalproex effectively treated the mood symptoms, and based on self-report, all patients remained abstinent during the trial.

Both valproate and alcohol consumption are known to cause temporary elevations in liver function tests, and in rare cases, fatal liver failure (Sussman and McLain 1979; Lieber and Leo 1992). Therefore, the safety of valproate in the alcoholic population has been questioned because of the potential for hepatotoxicity in patients who are already at risk for this complication. However, recent preliminary evidence suggests that liver enzymes do not dramatically increase in alcoholic patients who are receiving valproate, even if they are actively drinking (Sonne and Brady 1999*a*). Thus, valproate appears to be a safe and effective medication for alcoholic bipolar patients.

### Naltrexone

Because evidence suggests that active drinking may worsen bipolar symptoms, it makes sense that medications designed to decrease alcohol consumption may be useful in bipolar alcoholics. Naltrexone (ReVia^™^) is an FDA-approved medication designed to decrease cravings for alcohol. Maxwell and Shinderman (2000) reviewed the use of naltrexone in the treatment of alcoholism in 72 patients with major mental disorders, including bipolar disorder and major depression. Eighty-two percent of patients stayed on naltrexone for at least 8 weeks, 11 percent discontinued the medication because of side effects, and the remaining 7 percent discontinued for other reasons. The authors concluded that naltrexone was useful in treating patients with comorbid psychiatric and alcohol problems. However, Sonne and Brady (2000) reported on two cases of bipolar women (both actively hypomanic) who received naltrexone for alcohol cravings, and both had significant side effects similar to those of opiate withdrawal. Given that there is only preliminary data on the use of naltrexone in bipolar alcoholics to date, naltrexone should be used with caution in patients who have been actively hypomanic.

### Compliance

Medication compliance is an important issue to consider when assessing the effectiveness of medications. One study of the lifetime medication compliance of lithium and valproate in 44 alcohol and other drug-abusing bipolar patients found that patients were significantly more likely to take valproate (50 percent compliant) compared with lithium (21 percent compliant). Side effects, including lethargy, weight gain, and tremors, were listed as the main reason for non-compliance with lithium (Weiss et al. 1998). However, it is also important to note that prescription bottles for lithium usually have a warning label on them not to drink alcohol while taking the medication. Thus, if an alcoholic has the choice between taking lithium or drinking alcohol, it is very likely the alcoholic will not be compliant with lithium. Increased medication compliance with valproate may be an important factor in selecting a mood stabilizer for alcoholic bipolar patients.

### Psychosocial Interventions

Psychosocial interventions have often been considered the mainstays of treatment for alcoholism and other substance use disorders. Several studies have demonstrated success with cognitive behavioral therapy in treating alcoholism (Project MATCH Research Group 1998). Many of the principles of cognitive behavioral therapy are commonly applied in the treatment of both mood disorders and alcoholism. Weiss and colleagues (1999) have developed a relapse prevention group therapy using cognitive behavioral therapy techniques for treating patients with comorbid bipolar disorder and substance use disorder. This therapy uses an integrated approach; participants discuss topics that are relevant to both disorders, such as insomnia, emphasizing common aspects of recovery and relapse.

Interestingly, the same investigators (Weiss et al. 2000) evaluated the progress of a group of substance abusers with comorbid bipolar spectrum disorders who were pursuing psychosocial treatment independently, rather than as a result of being assigned to it by the researchers. Potential study participants were told that the investigators were interested in better understanding the relationship between bipolar disorder and substance abuse and therefore wished to see them monthly for 6 months. The investigators found that psychotherapy and Alcoholics Anonymous (AA) attendance decreased over time and that substance use tended to increase from month 1 to month 6. The focus of the study participants’ psychotherapy also changed, with less emphasis on their specific disorders and more emphasis on family, school, work, and other personal issues. Although differences in mood or substance use between months 1 and 6 were not statistically significant, there was a trend for increased substance use. If the study participants had continued with AA and if psychotherapy had continued to focus on bipolar disorder and alcoholism, the patients’ substance use might have improved. Given the generally poor prognosis associated with bipolar disorder and alcoholism, it is particularly important to continuously educate patients concerning the relationship between these two disorders. The authors concluded that the development of dually focused psychosocial treatments for this population may help improve substance use and affective outcomes.

## Conclusion

Bipolar disorder and alcoholism commonly co-occur. In two epidemiologic survey studies, alcohol dependence was more likely to occur with bipolar disorder than with all other psychiatric disorders except antisocial personality disorder. The nature of the relationship between alcoholism and bipolar disorder is complex and not well understood. It appears that alcohol use may worsen the clinical course of bipolar disorder, making it harder to treat. There is also evidence for a genetic link between the two conditions. Bipolar disorder complicated by alcoholism is associated with an increased number of hospitalizations, more mixed mania, earlier age of onset of bipolar disorder, and more suicidal ideation. Given the prevalence and morbidity of these two disorders, it is important to screen for substance abuse in all bipolar patients and to treat aggressively. Unfortunately, there has been little study of the appropriate treatment of this comorbidity. Several studies suggest that mood stabilizers (particularly valproate) may work better than lithium in treating alcoholic bipolar patients, but head-to-head comparison of lithium and valproate has not been carried out. Further study of this important comorbidity is needed to better understand its course and treatment.
